# Compliance with Australian stroke guideline recommendations for outdoor mobility and transport training by post-inpatient rehabilitation services: An observational cohort study

**DOI:** 10.1186/s12913-015-0952-7

**Published:** 2015-07-29

**Authors:** Annie McCluskey, Louise Ada, Patrick J. Kelly, Sandy Middleton, Stephen Goodall, Jeremy M. Grimshaw, Pip Logan, Mark Longworth, Aspasia Karageorge

**Affiliations:** Discipline of Occupational therapy, Faculty of Health Sciences, The University of Sydney, Sydney, NSW Australia; Discipline of Physiotherapy, Faculty of Health Sciences, The University of Sydney, Sydney, NSW Australia; Sydney School of Public Health, The University of Sydney, Sydney, NSW Australia; Nursing Research Institute, St Vincent’s Health Australia (Sydney) and Australian Catholic University, Level 5, DeLacey Building, St Vincent’s Hospital, Darlinghurst, NSW 2010 Australia; Centre for Health Economics Research and Evaluation, University of Technology Sydney, Sydney, NSW Australia; Ottawa Health Research Institute and Department of Medicine, Clinical Epidemiology Program, University of Ottawa, Ottawa, Canada; University of Nottingham, England, UK; NSW Agency for Clinical Innovation, Sydney, NSW Australia

**Keywords:** Physical therapy, Occupational therapy, Physiotherapy, Knowledge translation, Walking

## Abstract

**Background:**

Community participation is often restricted after stroke, due to reduced confidence and outdoor mobility. Australian clinical guidelines recommend that specific evidence-based interventions be delivered to target these restrictions, such as multiple escorted outdoor journeys. The aim of this study was to describe post-inpatient outdoor mobility and transport training delivered to stroke survivors in New South Wales, Australia and whether therapy differed according to type, sector or location of service provider.

**Methods:**

Using an observational retrospective cohort study design, 24 rehabilitation service providers were audited. Provider types included outpatient (*n* = 8), day therapy (*n* = 9), home-based rehabilitation (*n* = 5) and transitional aged care services (TAC, *n* = 2). Records of 15 stroke survivors who had received post-hospital rehabilitation were audited per service, for wait time, duration, amount of therapy and outdoor-related therapy.

**Results:**

A total of 311 records were audited. Median wait time for post-hospital therapy was 13 days (IQR, 5–35). Median duration of therapy was 68 days (IQR, 35–109), consisting of 11 sessions (IQR 4–19). Overall, a median of one session (IQR 0–3) was conducted outdoors per person. Outdoor-related therapy was similar across service providers, except that TAC delivered an average of 5.4 more outdoor-related sessions (95 % CI 4.4 to 6.4), and 3.5 more outings into public streets (95 % CI 2.8 to 4.3) per person, compared to outpatient services.

**Conclusion:**

The majority of service providers in the sample delivered little evidence-based outdoor mobility and travel training per stroke participant, as recommended in national stroke guidelines.

**Trial registration:**

Australian and New Zealand Clinical Trials Registry ACTRN12611000554965.

## Background

### Community participation and outdoor mobility after stroke

Community participation after a stroke is often greatly reduced when compared with adults in the general population. Stroke survivors in New Zealand reported an ongoing loss of confidence and fear of going out alone, resulting in social isolation [1]. A recent analysis of 776 stroke survivors in England and Wales found that 51 % were not ‘getting out and about as much as they would like’ [2]. Furthermore, that proportion did not change greatly between one and five years post-stroke. Reduced participation and social isolation seem to persist longer-term.

Reduced walking capacity is one reason why stroke survivors do not go out as often as they would like [1, 3]. Indoor walking practice, which is typically provided by many rehabilitation programs, does not automatically lead to improved outdoor walking or outings. For example, unsupported treadmill training significantly improved distance walked, speed and quality of life compared with controls [4], but did not translate into more outings. Stroke survivors have indicated a need for more ‘real-world’ practice in environments where they lack confidence: on ramps, escalators and in shopping malls [1].

### Interventions that improve community participation and outdoor mobility

Post-inpatient rehabilitation that includes multiple escorted walking trips in real-world environments can improve outings and community participation after stroke. In Australia and elsewhere, public and privately funded rehabilitation therapists may address this need, but so too may social service and charitable organisations, services for people with visual impairment and non-therapists. Logan and colleagues [5] employed occupational therapists to escort stroke survivors on multiple walking trips around local streets and to community venues, in addition to bus trips and help with return to driving. The most common component of this occupational therapy-led program was escorted walking trips to local parks and shops, which typically involved road crossings and kerb practice [6]. At the beginning of that trial [5], two-thirds of the stroke survivors were not getting out of the house as often as they wanted. The experimental group received a median of six outdoor-related sessions over three months and doubled their outdoor journeys, with two thirds getting out as often as they wanted, compared with no improvements in the control group. Furthermore, these gains were maintained 10 months beyond the intervention. Based on that evidence, current Australian stroke guidelines recommend that:

*People faced with difficulties in community transport and mobility should…undertake tailored strategies such as multiple....escorted outdoor journeys (which may include practice crossing roads, visits to local shops, bus or train travel), help to resume driving, aids and equipment, and written information about local transport options/alternatives, p 88’* [7].

Clinical audits completed by the National Stroke Foundation of Australia measure adherence to guideline recommendations, but reveal little about the delivery of post-inpatient services [8]. For example, the 2012 audit did not report on the amount or content of therapy sessions delivered to individual stroke survivors post-inpatient rehabilitation, particularly outdoor mobility and transport training.

### Study aim and research questions

The aim of the current study was to investigate outdoor mobility and transport training delivered to stroke survivors in New South Wales, (NSW) Australia. The research questions were:

1. What is the amount and content of outdoor-related sessions delivered to stroke survivors in NSW?
2. Is there a difference in the number of outdoor-related sessions delivered according to type (outpatient, day hospital, home, transitional aged care), sector (public, private), or location of service provider (centre, home)?

## Methods

### Design

An observational, retrospective cohort study was conducted, using medical record audit data from services providing post-inpatient rehabilitation. Approval to conduct the research and audit medical records was obtained from the Sydney South West Area Health Service – Concord Repatriation General Hospital Human Research Ethics Committee (09/CRGH/223) as lead ethics committee, the University of Sydney (#12777) and 21 single site committees in NSW.

### The sample of service providers

All known service providers that delivered post-hospital rehabilitation (*n* = 80) in Sydney, Newcastle and two regional areas of NSW (Illawarra and Central Coast) were approached to participate. Service providers were eligible to participate if they employed at least one occupational therapist and one physiotherapist, and received at least 10 stroke referrals per year (the stroke must have occurred within the previous 12 months).

Service providers were categorised into: type of provider (outpatient, day therapy, home-based rehabilitation (HBR), or transitional aged care (TAC); location (centre-based or home-based); and sector of service provider (public or private). Each service type is described later in results.

### Data collection: audit of medical records

Medical records were identified for audit by service managers. Managers identified recently discharged stroke survivors who had sustained their stroke no more than 12 months prior to starting therapy with the service, and had been seen by an occupational therapist and/or physiotherapist from that service. Medical records were audited between July 2010 and November 2012. Twenty consecutive records were requested so that 15 records could be audited, assuming that some records would be unavailable.

Data describing the service provider and stroke survivor were extracted during the audit, including demographic information (age, sex, marital status, living situation), date, side and type of stroke (haemorrhage or infarct), stroke severity (calculated retrospectively using the Scandinavian Stroke Scale (SSS) (SSS; [9]), and dependency (calculated retrospectively using the Modified Rankin Scale; [10] at commencement of post-inpatient rehabilitation. Data describing the post-inpatient therapy were also extracted, including waiting time (days from inpatient to service commencement), type of therapy (physiotherapy, occupational therapy or both), duration of therapy, and number of sessions delivered. Outdoor-related sessions were categorised as either an outing (therapist escorted the stroke survivor on an outing beyond the garden gate or perimeter of the hospital/property into a public street), outdoor practice (therapist escorted the stroke survivor into the garden or hospital grounds, on steps or uneven ground within the property but not into a public street), or information provision about outings/preparation for an outing (e.g., discussion about return to driving or planning a bus trip).

Two members of the research team were trained to conduct the audits. A data dictionary was used to increase consistency. Auditors were trained until agreement was reached on definitions, and sufficient examples collected to guide the auditors. Data were extracted independently from 10 files by two auditors, and compared until consistency was achieved, but no formal study of inter-rater agreement was conducted.

### Data analysis

Data from medical record audits were summarised using proportions, mean (SD) and median (IQR) where appropriate. Mean differences between type, sector and location of service provider were calculated for the number of outdoor-related sessions, number of outings, number of outdoor practice sessions, and number of information sessions provided per stroke survivor. Outpatient service providers were used as the reference group for service type, because outpatient services were, and still are, the most common provider type in NSW. Since outcomes were counts, negative binomial GEE models were used to calculate the mean differences, along with corresponding p-values and 95 % confidence intervals. GEE models were used to adjust for the clustering of stroke survivors within service providers, using an exchangeable correlation structure and robust standard errors calculated by the sandwich estimator [11]. Differences between groups were considered statistically significant if *p* < 0.05. Analyses were conducted in Stata 13 [12]. A secondary analysis was conducted which also adjusted for severity (mRS), age, gender and the natural logarithm of time post-stroke to confirm results after adjusting for potential confounders.

## Results

### Characteristics of service providers and stroke survivors

Of 80 hospital and community healthcare providers who were contacted, 32 met the eligibility criteria, 24 were recruited and eight declined (four publicly funded out-patient services and four private day therapy services).

Outpatient services (*n* = 8) were located in a hospital building, and employed several allied health disciplines including occupational therapists, physiotherapists and speech pathologists. This type of provider is common in NSW, with most public hospitals providing an outpatient service for recently discharged patients. Day therapy services (*n* = 9) were also centre-based, and employed several allied health disciplines. Each patient visit had to involve at least two allied health disciplines. A geriatrician or rehabilitation specialist typically led day therapy services. Five of the day therapy services were funded by private health insurance. Therapists provided most consultations onsite at the centre, with home and community visits conducted from time to time. HBR services (*n* = 5) delivered home and community-based therapy, and were jointly funded by state and commonwealth governments. TAC services (*n* = 2) also delivered home and community-based therapy for a maximum of 12 weeks, to older adults aged 65 or older at the conclusion of a hospital admission who had ongoing rehabilitation goals, and were often at risk of early admission to residential care. TAC services were funded by the Commonwealth government, and implemented by state agencies. HBR and TAC services employed several allied health disciplines, and mostly visited stroke survivors at home, although their office may have been located in a hospital building.

Of the 24 service providers recruited, 17 were categorised as centre-based (outpatient = 8; day therapy = 9), while seven were categorised as home-based (HBR = 5; TAC = 2). All 24 service providers were metropolitan. Five of the nine day therapy services were privately funded. A median of three physiotherapists and occupational therapists were employed per provider (range 2 to 13). A total of 311 medical records were audited. Services providers delivered a median of 15 medical records for audit (range 5–20).

Characteristics of the stroke survivors are presented in Table 1. Overall, the majority lived with a spouse/family, were 50 days post-stroke when they commenced post-inpatient therapy, typically walked independently indoors, and had a mild-moderate level of disability (median mRS 3, IQR 2 to 3). They waited 13 days to begin post-inpatient therapy, which comprised 11 sessions per stroke survivor over 2 months. The majority of stroke survivors received both physiotherapy and occupational therapy services. Of all therapy sessions provided, the largest proportion were categorised as mobility training (60 %), with fewer sessions provided for upper limb training (21 %), assessment (10 %), activities of daily living training (4 %) cognitive training (3 %) or ‘other’ activities (2 %).

**Table 1:** Characteristics of stroke survivors (*n* = 311) by type, sector and location of service provider

Characteristic	All service providers	Type of service provider				Sector of service provider		Location of service provider	
	(*n* = 311)	OP(*n* = 84)	DT(*n* = 117)	HBR(*n* = 76)	TAC(*n* = 34)	Public (*n* = 235)	Private (*n* = 76)	Centre (*n* = 201)	Home (*n* = 110)
Age (yr), mean (SD)	68 (15)	63 (15)	68 (17)	67 (16)	76 (10)	67 (16)	69 (17)	66 (15)	70 (13)
Sex, n male (%)	166 (72)	47 (56)	60 (51)	46 (61)	13 (38)	125 (53)	41 (54)	107 (53)	59 (54)
Marital status, n (%)									
Single	46 (16)	16 (19)	19 (16)	9 (12)	2 (6)	38 (18)	8 (12)	35 (19)	11 (11)
Married	180 (63)	55 (66)	62 (53)	44 (58)	19 (56)	138 (64)	42 (61)	117 (64)	63 (62)
Divorced	16 (6)	2 (2)	4 (3)	8 (11)	2 (6)	13 (6)	3 (4)	6 (3)	10 (10)
Widowed	44 (15)	4 (5)	22 (19)	7 (9)	11 (32)	28 (13)	16 (23)	26 (14)	18 (18)
Living situation, n (%)									
Alone	62 (21)	14 (17)	25 (21)	13 (17)	10 (30)	42 (18)	20 (26)	39 (21)	23 (22)
Family/spouse	228 (78)	62 (74)	84 (72)	59 (78)	23 (68)	176 (75)	52 (68)	146 (79)	82 (77)
Time post-stroke (days), med (IQR)	50 (31–92)	85 (40–157)	57 (37–88)	35 (15–67)	38 (19–46)	49 (26–94)	54 (34–82)	61 (38–112)	35 (17–63)
Side of stroke, n (%)									
Left	149 (48)	43 (51)	56 (48)	33 (43)	17 (50)	114 (49)	35 (46)	99 (49)	50 (46)
Right	120 (39)	34 (41)	47 (40)	24 (32)	15 (44)	87 (37)	33 (43)	81 (40)	39 (36)
Bilateral/unknown	42 (13)	7 (8)	14 (12)	19 (25)	2 (6)	34 (14)	8 (11)	21 (10)	21 (19)
Type of stroke, n (%)									
Infarct	127 (41)	36 (43)	50 (43)	23 (30)	18 (53)	97 (41)	30 (40)	86 (43)	41 (37)
Haemorrhage	49 (16)	16 (19)	18 (15)	12 (16)	3 (9)	32 (14)	17 (22)	34 (17)	15 (14)
Not stated	134 (43)	31 (37)	49 (42)	41 (54)	13 (38)	105 (45)	29 (38)	80 (40)	54 (49)
Stroke severity (SSS), mean (SD)	52 (4)	51 (4)	51 (4)	53 (3)	52 (3)	52 (4)	52 (4)	51 (4)	53 (3)
Dependency (mRS), med (IQR)	3 (2–3)	3 (2–3)	3 (2–3)	2 (2–3)	3 (3–3)	3 (2–3)	2 (2–3)	3 (2–3)	3 (2–3)
0–1, n (%)	20 (8)	3 (5)	10 (10)	6 (13)	1 (3)	13 (7)	7 (12)	13 (8)	7 (9)
≥2, n (%)	225 (92)	63 (96)	89 (90)	40 (87)	33 (97)	174 (93)	51 (88)	152 (92)	73 (91)
Post-inpatient therapy received									
Wait time (days), med (IQR)	13 (5–35)	26 (14–76)	14 (6–41)	8 (5–18)	3 (1–5)	13 (5–35)	12 (5–31)	21 (7–49)	6 (2–12)
Type, n (%)									
Physiotherapy only	64 (28)	25 (48)	15 (13)	24 (37)	0(0)	54 (35)	10 (13)	40 (24)	24 (37)
Occupational therapy only	37 (16)	18 (35)	13 (11)	6 (9)	0(0)	29 (19)	8 (11)	31 (19)	6 (9)
Both	130 (56)	9 (17)	86 (75)	35 (54)	34(100)	72 (46)	58 (76)	95 (57)	35 (54)
Duration (days), med (IQR)	68 (35–109)	98 (41–246)	57 (28–90)	56 (34–103)	80 (49–83)	74 (38–128)	57 (28–84)	69 (35–128)	62 (38–84)
Sessions (number), med (IQR)	11 (4–19)	8 (4–17)	12 (7–21)	5 (3–12)	19 (14–23)	8 (4–17)	14 (10–24)	11 (5–20)	10 (4–19)

*OP* outpatient, *DT* day therapy, *HBR* home-based rehabilitation, *TAC* transitional aged care, *mRS* modified Rankin Scale, 0–5, *SSS* scandinavian stroke scale, 0–60. *Time post-stroke* number of days between stroke (or hospital admission) and first contact with a therapist, *Wait time* Days between hospital discharge and first contact with a therapist

Across the service providers (Table 1), TAC saw more female participants who were slightly older and who lived alone. However, the level of disability and dependence were similar across all service providers. At the beginning of post-inpatient therapy, participants of centre-based providers were later post-stroke than participants of home-based providers. This difference was in part explained by the long wait (21 days) post-discharge for therapy, compared with six days for participants of home-based service providers. In the private sector, more participants received both physiotherapy and occupational therapy than the public sector. In the public sector, participants received fewer sessions over a longer period of time than the private sector.

### Outdoor-related therapy

Table 2 presents the outdoor-related therapy delivered to all stroke survivors. A median of one outdoor related session was delivered per participant (IQR 0 to 3). Escorted outings into a public street, use of public transport, and outdoor practice (such as walking in the hospital grounds or home garden) occurred rarely.

**Table 2:** Outdoor-related therapy sessions delivered per stroke survivor by type of service provider, mean difference (95 % CI) and statistical significance (p) between type of service provider

Therapy	All	Type of service provider				Mean difference between types of service provider^a^			*P* ^*^^*^
	(*n* = 311)	OP(*n* = 84)	DT(*n* = 117)	HBR(*n* = 76)	TAC(*n* = 34)	DT relative to OP	HBR relative to OP	TAC relative to OP	
Outdoor-related sessions per stroke survivor (number)									
Mean (SD)	2.1 (3.1)	1.4 (2.2)	1.4 (2.1)	2.0 (2.7)	6.8 (4.6)	0.0 (−0.7 to 0.6)	0.5 (−0.6 to 1.6)	5.4 (4.4 to 6.4)	<0.001
Med (IQR)	1 (0–3)	0 (0–2)	1 (0–2)	1 (0–3)	6 (3–11)				
Outings (number)									
Mean (SD)	0.9 (2.1)	0.6 (1.3)	0.3 (0.8)	0.9 (1.7)	4.1 (4.1)	−0.3 (−0.5 to 0.0)	0.4 (−0.1 to 0.8)	3.5 (2.8 to 4.3)	<0.001
Med (IQR)	0 (0–1)	0 (0–0)	0 (0–0)	0 (0–1)	3 (0–7)				
Outdoor practice (number)									
Mean (SD)	0.9 (1.7)	0.6 (1.7)	0.9 (1.7)	0.7 (1.3)	2.0 (2.0)	0.3 (−0.4 to 0.9)	0.1 (−0.60.2 to 0.7)	1.4 (0.6 to 2.2)	0.01
Med (IQR)	0 (0–1)	0 (0–0)	0 (0–1)	(0–1)	2 (0–3)				
Outdoor information (number)									
Mean (SD)	0.3 (0.7)	0.3 (0.7)	0.3 (0.6)	0.4 (0.8)	0.7 (1.0)	0.0 (−0.2 to 0.2)	0.1 (−0.2 to 0.4)	0.5 (0.0 to 0.9)	0.02
Med (IQR)	0 (0–0)	0 (0–0)	0 (0–0)	0 (0–1)	0 (0–1)				

*OP* outpatient, *DT* day therapy, *HBR* home-based rehabilitation, *TAC* transitional aged care

^a^Mean differences calculated from negative binomial GEE model

^*^^*^ *P* value for any difference between the four groups

### Difference in outdoor-related therapy between type, sector and location of service provider

Outdoor-related therapy per participant was similar across service providers, except that TAC delivered an average of 5.4 more outdoor-related sessions (95 % CI 4.4 to 6.4), and 3.5 more outings into public streets per participant (95 % CI 2.8 to 4.3) compared to outpatient services (Table 2). Smaller differences were seen between public and private sector providers, see Table 3. The largest difference was in number of outings per participant, with the public sector providing on average 0.7 more outings per stroke survivor (95 % CI 0.0 to 1.3, *p* = 0.01). Home-based service providers delivered an average of 1.8 more outdoor-related sessions (95 % CI 0.0 to 3.6, *p* = 0.007) and 1.3 more outings (95 % CI 0.2 to 2.5, *p* = 0.01) per participant than centre-based services (Table 4). Adjusting for severity (mRS), age, gender and time post-stroke did not qualitatively change results (results not shown; adjusted analyses conducted with *n* = 222 due to missing values).

**Table 3:** Outdoor-related therapy sessions delivered per stroke survivor by sector of service provider, mean difference (95 % CI) and statistical significance (p) between sectors of service provider

Therapy	Sector of service provider		Mean difference between sectors of service provider^a^	*P*
	Private	Public	Public relative to private	
	(*n* = 76)	(*n* = 235)		
Outdoor-related sessions per stroke survivor (number)				
Mean (SD)	1.5 (2.2)	2.3 (3.3)	0.6 (−0.4 to 1.6)	0.18
Med (IQR)	1 (0–2)	1 (0–3)		
Outings (number)				
Mean (SD)	0.3 (1.0)	1.1 (2.3)	0.7 (0.0 to 1.3)	0.01
Med (IQR)	0 (0–0)	0 (0–1)		
Outdoor practice (number)				
Mean (SD)	0.9 (1.8)	0.8 (1.7)	−0.2 (−0.7 to 0.4)	0.60
Med (IQR)	0 (0–1)	0 (0–1)		
Outdoor information (number)				
Mean (SD)	0.2 (0.5)	0.4 (0.8)	0.2 (−0.1 to 0.4)	0.25
Med (IQR)	0 (0–0)	0 (0–1)		

^a^Mean differences calculated from negative binomial GEE model

**Table 4:** Outdoor-related therapy sessions delivered per stroke survivor by location of service provider, mean difference (95 % CI) and statistical significance (p) between location of service provider

Therapy	Location of service provider		Mean difference between locations of service provider^a^	*P*
	Centre-based (*n* = 201)	Home-based (*n* = 110)	Home-based relative to centre-based	
Outdoor-related sessions per stroke survivor (number)				
Mean (SD)	1.4 (2.1)	3.5 (4.1)	1.8 (0.0 to 3.6)	0.007
Med (IQR)	1 (0–2)	2 (0–5)		
Outings (number)				
Mean (SD)	0.4 (1.0)	1.9 (3.0)	1.3 (0.2 to 2.5)	<0.001
Med (IQR)	0 (0–0)	0 (0–3)		
Outdoor practice (number)				
Mean (SD)	0.7 (1.7)	1.1 (1.6)	0.3 (−0.3 to 0.9)	0.35
Med (IQR)	0 (0–1)	0 (0–2)		
Outdoor information (number)				
Mean (SD)	0.3 (0.6)	0.5 (0.9)	0.2 (0.0 to 0.5)	0.06
Med (IQR)	0 (0–0)	0 (0–1)		

^a^Mean differences calculated from negative binomial GEE model

## Discussion

The key finding of this study was that very little therapy targeting outdoor mobility was delivered post-inpatient to stroke survivors, compared with the national guideline recommendation. Although the majority of sessions involved mobility training, only one session per stroke survivor was outdoor-related, with outings beyond the boundary of the centre or property occurring rarely. Two TAC services delivered more outings and outdoor practice per participant than other providers, demonstrating that this type of intervention can be delivered using existing resources.

### Few outdoor-related therapy sessions were provided per stroke survivor

Australian stroke guidelines recommend that people who have difficulty with community mobility and transport should receive interventions such as practice crossing roads, visiting local shops, travelling on buses and trains, help to resume driving, aids and equipment, and written information about transport options [7]. Most stroke survivors recruited to our sample had difficulty walking confidently outdoors, although many could walk independently indoors, based on their mRS score. Up to 60 % of therapy sessions addressed mobility, and were mostly provided by physiotherapists, but few outdoor-related sessions were provided. Although only two TAC service providers participated, and their results may not reflect other TAC services, they were able to deliver a mean of seven ‘outdoor-related’ sessions per participant; four of these seven sessions involving an actual ‘outing’ into a public street. These outings were provided by a physiotherapist, occupational therapist and/or therapy assistant. The other three sessions included outdoor practice on steps or uneven ground within the person’s garden or in hospital grounds, typically by a physiotherapist, or information provision about bus travel or community services, typically provided by an occupational therapist.

The above finding indicates that the national guideline recommendation can be achieved in practice. The number of sessions and dose of intervention were also consistent with the amount of outdoor-related sessions delivered per stroke survivor during a randomised controlled trial [5, 6]. The other service providers mostly trained mobility indoors, on flat linoleum floors with minimal perturbations, on a treadmill or stairs. While these sessions help *prepare* a person for outings, they do not simulate the demands of a real outing.

### Barriers and enablers to providing outdoor-related therapy sessions

Findings are consistent with an earlier smaller study, involving only public sector and metropolitan service providers in NSW [13]. Although the evidence for outdoor-related mobility training has been available since 2004, the intervention does not appear to have been translated into routine practice in NSW, Australia. Barriers to delivering the intervention have been investigated [14]. Therapists perceived that many stroke survivors did not expect nor want to go out soon after discharge home. Stroke survivors were afraid of falling and lacked confidence, which influenced what therapists did. Family members also influenced what interventions therapists offered, by discouraging outdoor journeys, preferring to drive their relatives to appointments rather than risk a fall during a bus trip or walk to the park. Other barriers included therapists’ doubts about their own and the ability of the team, to deliver multiple outings, and whether the intervention was compatible with their role. Finally, lack of knowledge about the intervention and the research evidence, as well as lack of skill and confidence in delivering the intervention inhibited best practice.

Findings suggest that specific strategies are needed to change perceptions and practice behaviours, and enable rehabilitation therapists to implement national guideline recommendations. The 2006 paper by Logan and colleagues [6] represents a good starting point, with a description of mobility-related goals and how to deliver this complex intervention. TAC service providers could also teach other therapists what to do. When questioned, they describe a process of the physiotherapist escorting a person to the garden gate, or beyond the property into a public street, then walking further to a park or bus stop on a second outing. They may practice negotiating kerbs, pavements and rough ground near the person’s home. An occupational therapist or assistant often becomes involved at that point, and may escort the stroke survivor out on a bus or shopping trip. What is unclear is how they actually changed their habits and routines, and what intervention they stopped providing in order to start providing more escorted outings.

Another potential enabler is use of not-for-profit, charitable or social service organisations, and non-therapists to provide escorted outings. Guidelines recommendations do not state what profession should provide specific therapies, so that providers can decide how to implement a therapy locally. In some regions, people with low vision (in addition to other conditions such as a stroke) can apply for extra training with orientation and mobility instructors employed by Guide Dogs NSW (http://visionloss.org.au/techniques-for-neurological-vision-impairment/). Dedicated travel training services also exist in some regions, for example South West Community Transport Travel Training (http://swct.com.au/services-provided/travel-training/). That organisation provides multiple, 1:1 escorted outings to adults with a range of disabilities and disadvantage.

The rehabilitation service providers in this study were similar to those available for most Australian stroke survivors. For example, a recent national audit [8] found that stroke survivors were referred to TAC service provider after hospital discharge at a rate of 17 %, which is similar to our sample where 11 % of participants were referred to a TAC service provider. Although Table 1 reveals differences in demographics between providers (ie age, sex, and living situation), the level of disability and dependence of stroke survivors was similar. That is, all service providers were treating the same population. TAC service providers were not seeing participants with less disability or dependence.

### Long waiting times for post-inpatient therapy services

There were also differences in waiting times for therapy. Outpatient services had the longest waiting time but then provided the longest duration of therapy. Home-based therapy was delivered earlier than centre-based and the private sector delivered a larger amount of therapy than the public sector. The median waiting time for therapy services was 13 days across all providers, and longer for centre-based service providers than for home-based service providers. The waiting time for outpatient services in our study was over a month.

Such delays are unacceptable. Long waiting times for publicly funded health services are not uncommon, and are not limited to stroke rehabilitation. Anecdotally, rehabilitation service providers state that they limit program duration to help ration services, manage demand and shorten waiting lists. Potential consequences for stroke survivors of longer waiting times include loss of mobility gained in hospital, falls and a loss of confidence, and social isolation [2]. Over 50 % of first falls post-stroke occur in the two-month period post-inpatient [15, 16]. While physiotherapy and occupational therapy services may not eliminate falls, they can identify hazards in or around the home, modify the environment to increase safety, and help prevent some falls.

### Study limitations

As with all research, this study had limitations. First, of 32 services that met the eligibility criteria, eight services declined to participate. Of these eight, four were public sector outpatient service providers, and the other four were private day therapy services. Thus, both public and private sector services were represented in the non-participating services. Second, only two TAC service providers were represented. While they appear to be a more efficient service provider type, their data are limited. Third, the number of medical records audited per provider ranged from five to 20. The small data set collected from some providers may not represent actual practice. Fourth, other factors may account for therapy decisions and practices, such as cold or wet weather, or the time of year. These factors were not recorded or analysed. Stroke survivors’ preferences and therapists’ clinical reasoning may also account for some of the therapy decisions, yet represent evidence-based practice. Exploration of these factors was beyond the scope of the current study.

### Implications for research and practice

Further research is warranted to explore why service providers were not complying with the guideline recommendation, and what they know and think about the evidence. Also important to explore is how some providers were able to implement this evidence-based recommendation, and if other TAC service providers are currently delivering best practice. A qualitative study is being conducted at present by the lead author, exploring how successful teams changed their practice, managed their time and redesigned their work to provide escorted outings, and what motivated them to change. A cluster randomised controlled trial is also being completed by the authors, evaluating the efficacy of a behavior change implementation program with these teams [17]. The behavior change program includes a training workshop about delivery of escorted outings, tailored feedback about medical record audits from the current study, and barrier identification.

Practice implications include a need for feedback to service providers and policymakers about the variability in waiting times, number of sessions overall, and low number of outdoor-related sessions. Raising therapists’ awareness of community-based participation training, as opposed to indoor impairment-based training, is likely to be of benefit to community-dwelling stroke survivors. There is also potential for therapists to learn from providers (such as TAC) about how to make time for escorted outings, and how to progress sessions.

## Conclusions

In conclusion, these service providers of post-inpatient therapy in NSW Australia were not providing evidence-based practice and outdoor mobility training to stroke survivors with mobility restrictions, as recommended in national stroke guidelines. Further study is needed into how service providers successfully deliver more escorted outings.

## Abbreviations

OP: Outpatient
DT: Day therapy
HBR: Home based rehabilitation
TAC: Transitional aged care
NSW: New South Wales (Australia)
SSS: Scandinavian Stroke Scale
mRS: Modified Rankin Scale
IQR: Interquartile range
CI: Confidence interval
SD: Standard deviation
